# Diseases of the Stomach

**Published:** 1895-10-05

**Authors:** 


### Progress in Medicine.

### DISEASES OF THE STOMACH.

(*Continued from page 447, Vol. XVIII*)

**Merycismus.**—The explanation of the occasional occurrence of rumination or remastication in human beings is doubtful. Hammond wrote last year that only about fifty cases had been reported altogether, but many more probably exist. He described an instance where a cure followed upon trephining carried out for another purpose. In a second patient he found that there was no evidence of dilatation of the stomach or failure of absorption or secretion. On the whole, he is not inclined to believe the condition to be due to a neurosis, but that it is rather an instance of atavism, or reversion to an earlier type, in which rumination was a normal process.[1]

**Stenosis of the Pylorus**, according to Max Einhorn, is usually marked by much vomiting and frequent pains, great loss of weight, and constant ischochymia, food such as rice, which has been taken the previous night, being found in the stomach next day. In the malignant form there is generally constant pain; the illness is of less duration. The presence of lactic acid, and the absence of H Cl is also to be noted. On the other hand, in chronic gastric atony and catarrh, any ischochymia disappears after a few washings of the stomach. As to treatment, if no operation can be attempted, small, frequent meals, lavage, and spraying the stomach with nitrate of silver, and rectal injections of pure water if thirst be present, afford much relief.[2]

**Dilatation of the Stomach.**—Matthieu has found that when this affection is due to spasm of the pylorus, warm drinks and alkalies are useful. When, as is perhaps more frequently the case, muscular atony has been the cause, we should employ, in addition, ipecacuanha, strychnia, massage, and electricity. Concentrated food must be given in a finely divided state at long intervals, so that few meals are taken in the twenty-four hours. The stomach may be washed out once a week to prevent fermentation, and antiseptics employed for the same end.[3] Salzer and Osler mention the presence of movable, or at least palpable kidney as an indication of dilated stomach.[4] Soltau Fenwick, at the Clinical Society, described a fatal form of tetany associated with chronic dilatation. One case was fatal after the seventh attack of the spasms, and the other recovered under the use of lavage twice a week. Some thirty cases have now been published, and in the majority the cause of the dilatation was found to be a chronic ulcer in the vicinity of the pylorus. Severe vomiting in all preceded the tetany, and about 70 per cent. of the patients died. The best treatment appears to be regular lavage with warm water or a weak solution of resorcin.[5] The peristaltic movements of the stomach which occur normally during digestion alone may go on at other times. This has been regarded as a mark of pyloric stenosis, but Harnot shows that it often happens without stenosis, nor is stenosis always accompanied by it. In fact, the true pathology is unknown.[6] The presence of acetone was put forward by Lorenz as a test of primary disease of the stomach, but his results are negatived by those of Savelieff, who has shown that it is very rarely met with.[7] Again, sulphuretted hydrogen is frequently present in benign dilatation, though not in malignant disease according to Boas.[8] Even the presence of much free H Cl does not prevent its formation in dilated stomachs.[9] Rachford has endeavoured to show that many headaches, epilepsies, and gastric neuroses are due to leucomain (and chiefly to paraxanthin) poisoning. Paraxanthin is not formed in the kidney, and cannot normally be demonstrated in less than four litres of urine, whereas during attacks in these cases it is excreted from the blood in large quantities, and produces, if injected into mice, convulsions and dyspnœa. The patients may notice sensations of flushing and burning before the attacks, and the urine becomes scanty, and shows a fall in the urea excreted, but the pulse remains strong throughout. It is not clear how the affection may be diagnosed from attacks due to other causes, unless the paraxanthin is isolated. Permanganate of potash may be given to neutralise it.[10] Basy also notices that attacks of pain in the stomach and vomiting occasionally replace for a time attacks of migraine without evidence of any stomach disease, and are probably due to the same cause as they are.[11] For the treatment of dilatation depending on cicatrices of the pylorus, Podies advises Loreta's operation of stretching the pylorus and scraping the floor of any ulcers found there, instead of gastro enterostomy.[12]

**Bicarbonate of Soda.**—Much discussion has taken place on the value of this substance in gastric affections. Reichman, from 103 experiments, claims that it in no way influences the secretory power of the stomach, though it may diminish acidity for the time.[13] Linoissier and Lemoine have repeated their experiments, and find that the effects of the drug are primarily stimulative of secretion, especially when given an hour before a meal; but if in excess, it may neutralise the results of the acid. This can only be shown by repeated tests during the whole course of digestion. The stimulation is followed, after prolonged administration of the drug, by depression of the secretion, or a sedative action through alkalisation of the blood. For hyperacidity, then, we may give large doses during or after a meal, continued for a long time, and for deficient action small doses beforehand for a short time. Other observers have possibly erred through making only one observation after a meal.[14] Rosenbach issued a warning against the dangers of soda treatment when prolonged as weakening the secretory power and permitting prolonged fermentation.[15] Matthieu found that 45 grains of soda given before a meal reduced the amount of H Cl actually secreted, and large doses of soda and magnesia in one case produced cystitis and hæmaturia, but Hayem mentioned that he had given 8 drachms daily of soda without ill effects.[16] Ferrand, to prevent ill effects, gives the subcarbonate with the bicarbonate.[17] Dujardin Beaumetz, and Gillespie have come round to the common view that, given beforehand it is a stimulant, and after meals a sedative with regard to the acidity of the stomach. The former gave it for dilatation during or after meals.[18] In opposition to the view of the danger of prolonged soda treatment, Taylor mentioned a patient who took tablespoonful doses for years with much relief,[19] and R. G. Feek, one who has actually consumed 2,200 lbs. in twenty-one years, and is now in vigorous health and taking 2 lbs. a week![20]

**Hyperacidity.**—A. L. Gillespie quotes Schreiber as arguing that acid is secreted even during fasting, and Martins as finding from ·04 to ·15 H Cl in the stomachs of sixteen healthy soldiers after a thirteen hours' fast, which is, however, negatived by the experiments of Strauss, who regards chronic secretion as pathological.[21]

Soltau Fenwick, in the treatment of hyperacidity, recommends careful moderate diet, chiefly nitrogenous, with large doses of alkalies, after meals, for long periods and small doses of liq. potassæ and bromide twice daily.[22] He regards chronic hypersecretion of H Cl as a final stage of hyperacidity. The urine is neutral and often scanty, the urea excessive, and the chlorides very deficient, while the quantity of fluid obtained from the stomach after a test is very large and H Cl is found in the fasting state. Dilatation and attacks of tetany may supervene, and frequent vomiting is commonly present.[23] Hayem asserts that there are no special cells for the secretion of H Cl and that in hypersecretion there is no increase in the parietal cells, while on the other hand the new-born child secretes no H Cl, though the parietal cells are well developed. He does not regard the absence of H Cl in cancer as pathognomic or important. When it occurs it is due to the atrophic gastritis so often present. Wiener and Mierzynsky advocate a volumetric[24] method for estimation of the H Cl by conversion into barium chromate and measuring the oxygen evolved,[25] while Friedenwald has examined the dimethylamido-azo-benzol test and finds it very simple, reacting to minute quantities of free acid, and not to organic acids. It is more sensitive than Gunsberg's and Boas' tests.[26] A few drops of a ·5 per cent. alcoholic solution added to a gastric filtrate give a red tinge if free acid is present. The resorcin test is cheap, simple, as reliable as phloroglucin, and keeps well according to Friedenwald. The formula is resorcin 5, sacch. alb 3, spiritus dil. 100. A few drops are evaporated with an equal quantity of filtrate, and produce a red ring if free HCl is present.[27]

In anæmia Oswald lays down that the dyspepsia does not depend on a lack of H Cl, and that it is rarely diminished in these cases, more often there is an excess. He explains the divergent results of other authors as due to the neglect of the combined acid and other errors.[28]

[1] Int. Med. Mag., Oct., 1894. [2] Med. Rec., Jan. 19, 1895. [3] B M. J., July 20, 1895. [4] New York Med. J., Jan. 12, 1895. [5] Lancet, Oct. 20, 1894. [6] Glasgow Med. J., Feb., 1895. [7] Am. M.S. Bullet., Oct., 1894. [8] Am. J. Med. Sc., June, 1895. [9] B. M. J., Feb. 2, 1895. [10] Med. Rec., June 22. [11] Am. J. Med. Sc., June, 1895. [12] B. M. J., July 20. [13] Practitioner, June. [14] Med. Chron., March. [15] Birm. Med. Rev., Feb. [16] Med Week, Aug. 3, 1894, and Mar. 29, 1895. [17] Med. Week. Ap. 5, 1895. [18] N. Y. Med. J., Nov. 3. [19] Glasgow Med. J., Nov., 1894. [20] Med. Rec., Sep. 22. [21] Edinb. Med. J., June, 1895. [22] Clin. J., March 13. [23] Clin. J., June 12. [24] Med. Rec.. May 25. [25] B. M. J., Ap. 20, 1895; Dec. 15, 1894. [26] Med. Rec., Ap. 6, 1895. [27] Med. Rec., Oct. [28] Birm. Med. Rev.. Feb.

